# The Effectiveness of Interventions for Increasing COVID-19 Vaccine Uptake: A Systematic Review

**DOI:** 10.3390/vaccines10030386

**Published:** 2022-03-03

**Authors:** Eleonore Batteux, Freya Mills, Leah Ffion Jones, Charles Symons, Dale Weston

**Affiliations:** 1 Behavioural Science and Insights Unit, UK Health Security Agency, Salisbury SP4 0JG, UK; e.batteux@ucl.ac.uk (E.B.); leah.jones@phe.gov.uk (L.F.J.); charles.symons@phe.gov.uk (C.S.); dale.weston@phe.gov.uk (D.W.); 2 Centre for the Study of Decision-Making Uncertainty, University College London, London WC1E 6BT, UK

**Keywords:** COVID-19, vaccines, vaccine uptake, systematic review, interventions, behaviour change

## Abstract

Vaccination is vital to protect the public against COVID-19. The aim of this systematic review is to identify and evaluate the effectiveness of interventions to increase COVID-19 vaccine uptake. We searched a range of databases (Embase, Medline, Psychology & Behavioral Science, PsycInfo, Web of Science and NIH Preprints Portfolio) from March 2020 to July 2021 for studies which reported primary quantitative or qualitative research on interventions to increase COVID-19 vaccine uptake. Outcome measures included vaccination uptake and reported intention to vaccinate. Reviews, position papers, conference abstracts, protocol papers and papers not in English were excluded. The NHLBI quality assessment was used to assess risk of bias. In total, 39 studies across 33 papers met the inclusion criteria. A total of 28 were assessed as good quality. They included interventions relating to communication content, communication delivery, communication presentation, policy or vaccination delivery, with 7 measuring vaccination uptake and 32 measuring vaccination intention. A narrative synthesis was conducted, which highlighted that there is reasonable evidence from studies investigating real behaviour suggesting that personalising communications and sending booking reminders via text message increases vaccine uptake. Findings on vaccination intention are mixed but suggest that communicating uncertainty about the vaccine does not decrease intention, whereas making vaccination mandatory could have a negative impact. Although much of the research used experimental designs, very few measured real behavioural outcomes. Understanding which interventions are most effective amongst vaccine-hesitant populations and in the context of booster vaccinations will be important as vaccine roll outs continue across the world.

## 1. Introduction

A key measure in the fight against COVID-19 is vaccination. Vaccination programmes were launched across the world from December 2020, with many countries having now vaccinated a substantial proportion of their population. At the time of writing, 62.8% of the global population have received at least one dose of the vaccine and 55.4% are fully vaccinated, although there are large discrepancies in vaccination rates between countries [1]. In the UK, 85% of the population aged 12+ have received the first two doses and 66.1% have received a booster dose [2]. Given that high rates of vaccination are required to ensure the population is adequately protected, and in light of the role of booster vaccination in combating the threat of new variants [3], it is necessary to identify how to effectively increase COVID-19 vaccine uptake.

Vaccine hesitancy is complex, with a plethora of underlying concerns. Some of these concerns pertain to vaccinations more broadly [4,5], whilst others are specific to the context of COVID-19. For example, it has been reported that there are concerns regarding the speed at which vaccines were developed [5], as well as worries regarding vaccine effectiveness, side-effects and safety of the COVID-19 vaccine [4,6]. In addition to barriers relating to knowledge and beliefs, there are also environmental barriers, such as vaccine shortages, inaccessible vaccination sites and ability to get time off work, which can serve as barriers even amongst the most willing [7,8]. Barriers are also not universal; therefore, it is important to understand the extent to which interventions are effective amongst specific groups. For example, some younger people do not perceive the vaccine to be necessary for those at low risk of harm from the virus [9]; pregnant women have specific concerns for their baby [10]; and asylum seekers may fear persecution [11].

In addition to understanding the barriers to vaccine uptake, it is also necessary to draw from published literature concerning effective interventions to identify what is likely to work for increasing vaccine intentions and behaviours. Existing interventions have included communication campaigns, incentivisation and reminders. It is important to understand the extent to which these interventions impacted vaccine uptake so that future interventions can build upon their successes and address their limitations. We conducted a systematic review to identify and evaluate the effectiveness of interventions which can increase COVID-19 vaccine uptake. Given that this review is taking place at a time where the published literature on interventions implemented during the vaccine rollout is still in its relative infancy, the scope of this review was broad and included global studies on either COVID-19 vaccination intention or behaviour.

## 2. Materials and Methods

The research methods of this review are reported following the Preferred Reporting Items for Systematic Review and Meta-Analysis (PRIMSA) statement [12] (the PRISMA checklist for each item is included in Supplementary Table S1). The protocol for this review was prospectively registered on PROSPERO, Internal Prospective Register of Systematic Reviews, registration number: CRD42021266943 (https://www.crd.york.ac.uk/prospero/display_record.php?RecordID=266943 (accessed on 14 January 2022)).

### 2.1. Eligibility Criteria

Studies were eligible if they were primary quantitative or qualitative research on interventions to increase COVID-19 vaccine uptake. Outcome measures included measures of uptake, either through self-reports, observations or vaccination records; measures of uptake intentions; and measures of vaccine hesitancy. Studies which explored attitudes towards vaccine uptake or the demographic determinants of vaccine uptake without reporting possible interventions were excluded as this review was primarily focused on identifying effective interventions. Published research and pre-publication articles were included. Reviews, position papers, conference abstracts, protocol papers, unpublished studies and studies published in languages other than English were excluded.

### 2.2. Search

A systematic search was conducted by UKHSA Knowledge and Library Services in July 2021 for papers between March 2020 and July 2021. Sources searched included Embase, Medline, Psychology & Behavioral Science, PsycInfo, Web of Science and NIH Preprints Portfolio. Search terms included terms related to COVID-19 (e.g., COVID-19, coronavirus, and SARS-CoV-2), vaccination (e.g., vaccination, immunisation, and uptake) and interventions (e.g., intervention, message, and behaviour change). A complete list of search terms is available in Supplementary Table S2. A Google search for relevant papers was also conducted by the study authors in July 2021 for any other relevant papers not returned by the search.

### 2.3. Study Identification

**Figure 1:**
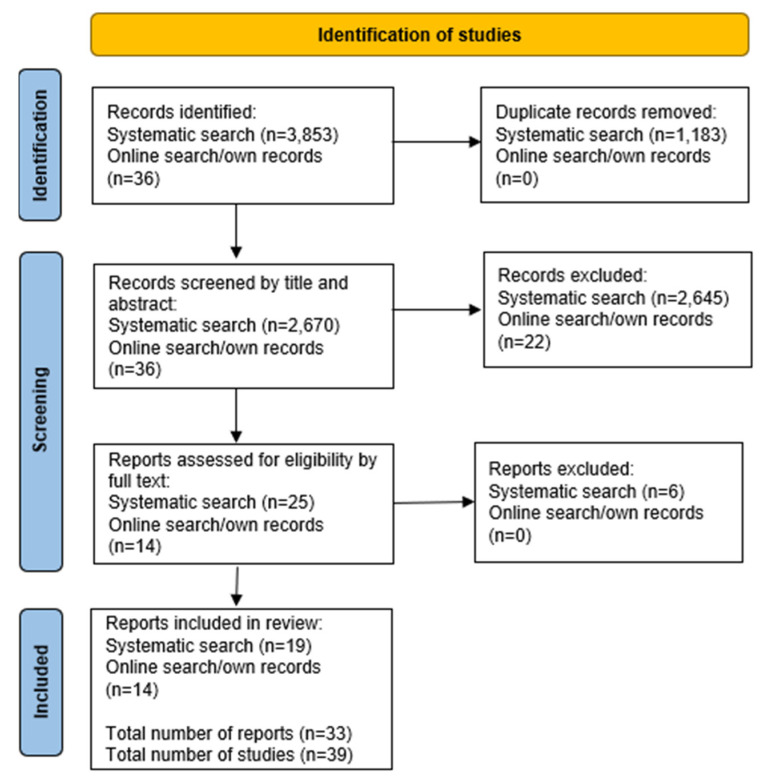
PRISMA flow diagram of the identification of studies.

The study identification process is detailed in Figure 1. FM and EB conducted initial title and abstract screening of all records on Rayyan, a review screening website [13]. Two of the reviewers (FM and EB) then screened the full text of relevant records, with each reviewer screening half of the records. The main reasons for exclusion were studies reporting the wrong outcome (e.g., flu vaccine uptake), studies not reporting an intervention or studies being the wrong publication type (e.g., review and protocol). After all texts were independently reviewed, the studies identified as eligible were then discussed by FM and EB to make sure they met the inclusion criteria, at which point a further six reports were excluded. At this stage, studies were excluded either because they focused on outcomes (e.g., vaccine misinformation) or methods (e.g., modelling) outside of our inclusion criteria. By the end of this process, 39 studies were deemed eligible and included in this review. A list of included studies with their methodological characteristics is available in Supplementary Table S3.

### 2.4. Data Extraction

Two of the reviewers (FM and EB) each independently extracted the data from half of the included studies. FM and EB checked the full extracted dataset for accuracy and completeness. They extracted the following data: author, year of publication, report type, study design, participant characteristics, recruitment method, time point of data collection, country of study, intervention type, intervention description, intervention comparison description, vaccine uptake measure, and effectiveness of intervention. All results relating to vaccination uptake or intentions were included.

### 2.5. Risk of Bias Assessment

The original protocol considered using RoB-2 for randomised studies and ROBIN-I for non-randomised studies, but the National Heart, Lung and Blood Institute (NHLBI) quality assessment tools were deemed more suitable for the included studies given there were case–control studies [14]. Two reviewers (FM and EB) conducted a risk of bias assessment on the included studies using the NHLBI tools for controlled intervention studies, cross-sectional studies and case–control studies, with each reviewer independently assessing half of the studies. Before assessing the quality of the studies, FM and EB discussed the criteria to ensure consistent rating. Each author then independently assessed the quality of half of the studies each, and classified each study as being of good, fair or poor quality. FM and EB then cross-assessed a further 10 studies, blinded to the first assessment made by the other reviewer, so that 50% of the studies had been independently assessed twice to ensure consistency in the risk of bias assessment. Any disagreements were resolved through discussion by FM and EB.

### 2.6. Data Synthesis

A systematic narrative synthesis was conducted which summarises the characteristics and findings of the included studies. Studies were categorised by type of intervention by two of the reviewers (FM and EB) and reviewed by the remaining reviewers (LJ, CS, and DW). The final categories were communication content, communication presentation, communication delivery, policy and vaccination delivery. Findings were interpreted by considering the study context, population, methodology and data quality. Due to the variability in interventions and outcomes, the reviewers did not compute summary statistics or effect size estimates.

When assessing the effectiveness of an intervention, the authors considered potential limitations, including outcome measures, study design, lack of comparators and publication status. When considering possible causes of heterogeneity amongst study results, the reviewers used the explanations provided by the study authors. Due to the limited number of papers for each type of outcome and intervention, the reviewers did not perform any formal analysis to assess heterogeneity.

## 3. Results and Discussion

A summary of the characteristics of each study can be found in Table 1 (full details in Supplementary Table S2). Across the 39 included studies, a range of interventions to increase COVID-19 vaccine uptake were reported relating to communication delivery, communication content, communication presentation, policy and vaccine delivery. In total, 32 studies measured vaccination intention and 7 measured vaccination behaviour (vaccination rates or scheduled appointments). Interventions were tested with a range of methods, most commonly online randomised controlled trials (RCTs) (20 studies) but also including field RCTs, online experiments (including discrete choice experiments, often used in health care [15]), quasi-experimental and cross-sectional studies. The included studies were tested in a range of countries, including the UK, the US, Germany, Japan, China, Hong Kong and Israel. Most studies were from the US (22 studies), with 9 studies from the UK. Findings are presented by type of intervention and outcome measure (real behaviour or intention).

### 3.1. Risk of Bias Assessment

The overall quality of the studies was good. For controlled intervention studies, 24 studies were rated as good and 7 as fair. For cross-sectional studies, 2 studies were rated as good, 1 as fair and 2 as poor. For case–control studies, 2 studies were rated as good and 1 as fair. The most common reason for a fair quality rating was because the studies did not report methodological details such as blinding and drop-out rates. The reason two studies were rated as poor quality was because differences in outcome pre- and post-intervention for cross-sectional studies without a control were inferred from only one measure taken after the intervention. The risk of bias assessment for each study can be found in Supplementary Table S4.

### 3.2. Communication Content

#### 3.2.1. Benefits of Vaccination

##### Vaccination Behaviour

A field RCT (pre-print) found that communicating the personal and collective benefits of vaccination did not increase uptake. In particular, it found that text message booking reminders increased uptake regardless of their message content, including whether it referred to protecting oneself and others or to vaccination offering a fresh start after a tough year [16]. However, a further field RCT (pre-print) found that text messages communicating the personal or social benefit increased vaccination uptake [17].

##### Vaccination Intention

Studies testing whether communicating the personal and collective benefits of vaccination increases vaccination intention have yielded mixed findings. An online RCT found that a news story communicating the personal health risks of not getting vaccinated, and another communicating the collective public health consequences of not getting vaccinated, increased vaccination intention [18]. Another online RCT (pre-print), measuring a digital expression of interest, found that after watching a video promoting the health benefits of the COVID-19 vaccine, 16% of unvaccinated participants wanted more information [19]. This proportion of participants was similar to those who watched a video including the promotion of cash lotteries, although less effective than a video promoting states using cash vouchers. Another online RCT found that messages emphasising personal benefit, collective benefit or both did not affect vaccine hesitancy in their overall sample but did have effects among hesitant individuals, where emphasising personal benefit reduced vaccine hesitancy, as did combining personal and collective benefits, the seriousness of the pandemic and addressing safety concerns, with personal benefit leading to the greatest reduction in hesitancy [20]. They also found demographic differences, as Asian individuals showed a greater reduction in hesitancy when collective and personal benefits were combined, whilst men had less hesitancy than women in the personal benefits condition [20].

Other studies found that communicating the personal and collective benefits of vaccination did not increase uptake. An online RCT found that communicating rapid uptake of the vaccine as being important to reduce infections and protect others did not influence vaccination intentions [21]. Another online RCT (pre-print) found that a fact box communicating the risk of getting a COVID-19 vaccine compared to the risk of getting the COVID-19 virus had no impact on vaccination intention [22]. The authors suggest this could be because the fact box highlighted risks relating to the vaccine which participants paid more attention to or may not have previously been aware of. Finally, a news story communicating the economic costs of not getting vaccinated did not affect vaccination intention in an online RCT [18].

#### 3.2.2. Effectiveness and Safety

##### Vaccination Behaviour

Communicating the effectiveness and safety of the vaccine can increase uptake. A field experiment (pre-print) found that a booking reminder emphasising vaccine effectiveness was more effective than one emphasising social benefits and others having had the vaccine [17]. Another field RCT found that an email comparing the risks of the COVID-19 vaccine to the more severe risks of the virus increased uptake [23]. However, this intervention had a similar impact on uptake as an email focusing on social norms. The authors also acknowledged that it was difficult to isolate the effects of effectiveness and safety as both emails also had components of personalisation, scarcity and active choice.

##### Vaccination Intention

Research exploring the role of describing the safety and effectiveness of vaccines on vaccination intention is mixed. A study found that a message emphasising that the NHS declares the vaccine safe and effective increased vaccination intention [24]. This was echoed in an online experiment, particularly amongst vaccine-hesitant individuals [25]. However, neither study can identify whether safety or effectiveness had a greater effect. Similarly, an online RCT found that a news story about the vaccine being safe and effective increased vaccination intention [26]. However, another online RCT found that a range of interventions communicating effectiveness and safety were not more effective than the control [27]. Furthermore, another online experiment presented the risks of the COVID-19 vaccine in comparison to the risks of the COVID-19 virus and found that it did not increase vaccination intention [22]. Finally, two online RCTs tested whether communicating that the COVID-19 vaccine is more effective than the flu vaccine increased vaccination intention. One in the UK found that this was effective [25], whereas one in the US did not [28].

The level of effectiveness and safety of vaccines can impact vaccination intention. An online discrete choice experiment (DCE) found that decreasing effectiveness from 70% to 50% and decreasing duration of immunity from 6 to 3 months reduced vaccine uptake [29]. Similarly, a study (pre-print) found that vaccines with 95% efficacy are preferred to 50% or 70% efficacy [30]. However, an alternative study found no impact of news articles stating 80% vs. 20% effectiveness [31]. Relating to safety, a DCE found that increasing the risk of side-effects reduced intentions [29], while a cross-sectional study (pre-print) found that less risk of side-effects is preferable [30]. This cross-sectional study also found that a protection scheme (i.e., medical insurance) to compensate for side-effects can increase intentions.

#### 3.2.3. Vaccine Development

##### Vaccination Intention

Addressing concerns about the speed of vaccine development has mixed effects on vaccination intention. In an online RCT, doing so decreased vaccine hesitancy in strongly hesitant individuals [20]. Furthermore, in an online experiment (pre-print), participants were more likely to accept less effective vaccines if the development process took 12 rather than 7 months [32]. However, a message detailing the development process (pre-print) did not increase vaccination intention compared to no message in an online RCT in the US [22]. An online RCT found that a news story highlighting the rigours of clinical trials did not increase vaccination intention, whereas not highlighting the rigours of clinical trials did increase vaccination intention [18]. Finally, a news story highlighting that President Trump pushed for rapid approval of vaccines decreased intention [26].

#### 3.2.4. Social Norms

##### Vaccination Behaviour

Social norm interventions asking recipients to ‘join the millions’ being vaccinated can be effective. A field experiment (pre-print) found that a booking reminder emphasising to ‘join the 3.5 million vaccinated citizens’ increased vaccine uptake, but to a lesser extent than one emphasising the personal benefit of getting vaccinated [17]. Another field RCT with unvaccinated health care workers found that sending an email telling them that more than 11 million Americans, including many of their colleagues, had been vaccinated increased uptake [23]. However, this effect was similar to the effect of an email comparing the risks of the vaccine to the more severe risks of COVID-19.

##### Vaccination Intention

Emphasising that others will or have been vaccinated has variable effects on vaccination intention. In an online RCT, a news story about others being willing to get vaccinated increased intention, while a news story about others being unwilling to get vaccinated decreased intention [26]. In an online RCT (pre-print), participants who received accurate information about levels of vaccine acceptance showed increased vaccination acceptance, particularly amongst those unsure about receiving the vaccine and in certain countries (e.g., Pakistan and Vietnam) [33]. The authors suggest this could be due to people underestimating the extent to which others would get vaccinated at the time (October 2020). However, although communicating in an online RCT that 85% of people plan to take the vaccine was more effective than communicating that 45% of people plan to take the vaccine, neither were more effective than no information [24].

#### 3.2.5. Herd Immunity

##### Vaccination Intention

An online RCT found that a news story emphasising the necessary coverage rate of vaccination to achieve herd immunity increased vaccination intention compared to an unrelated news story [18]. Previous experimental evidence also suggests that communicating about herd immunity can increase vaccine uptake [34,35].

### 3.3. Communication Presentation

#### 3.3.1. Personalisation

##### Vaccination Behaviour

Personalising vaccination invitations can be effective. A field RCT (pre-print) found that text message booking reminders emphasising personalisation increased uptake [16]. A follow-up field RCT found that sending a second reminder increased uptake regardless of the message content, including whether it referred to the individual being one of the few to have early access to the vaccine [16].

##### Vaccination Intention

In an online experiment (pre-print), a text message booking reminder emphasising personalisation did not increase intention [16]. This is particularly noteworthy as an identical field RCT, run concurrently to the online experiment, found the opposite effect, as mentioned in the preceding section [16].

#### 3.3.2. Framing

##### Vaccination Intention

There was mixed effectiveness of framing on vaccination intention, although the evidence is limited. An online experiment (pre-print) found that positive framing of vaccine safety (95% safe) increased acceptance of less effective vaccines compared to negative framing (5% unsafe) [32]. On the other hand, an online RCT did not find a difference in intention when a news article framed vaccination protection against the virus negatively as opposed to positively [31]. An online RCT did not find a difference in intention when describing a vaccination lottery prize as a gain compared to a loss [36].

#### 3.3.3. Numerical Format

##### Vaccination Intention

An online RCT did not find a difference in vaccination intention between presenting effectiveness as a percentage or as a frequency [31]. An online RCT did not find a difference between presenting vaccination lottery winners as ‘5 total winners’ to ‘1 winner for each of the 5 weeks’ [36].

#### 3.3.4. Uncertainty

##### Vaccination Intention

Communicating uncertainty could increase intentions and protect existing intentions in the event of changes in information about the vaccine. An online RCT found some evidence that communicating uncertainty about COVID-19 increased vaccination intentions, which was also the case in the presence of an uncertainty-normalising intervention [37]. Two online RCTs did not find an effect of communicating uncertainty about vaccine effectiveness on vaccination intention compared to communicating with certainty [27,38]. In addition, communicating that there is uncertainty relating to effectiveness at the outset can mitigate a reduction in vaccination intention once conflicting information on effectiveness arises later on (pre-print) [38].

### 3.4. Communication Delivery

#### 3.4.1. Messenger

##### Vaccination Intention

There was mixed evidence for the effect of messengers on intention. An online DCE (pre-print) found that text message reminders were preferred if delivered by the NHS or one’s GP, rather than one’s best friend [39]. An online RCT (pre-print) found that a Republican endorsement of the vaccine through a video and short essay was more effective than a Democrat endorsement intentions among Republican or Republican leaning participants [40]. An online RCT found that a news story indicating that President Trump pushed vaccines for rapid approval decreased vaccination intention, regardless of party identification [26]. Finally, whether a newspaper opinion piece about the importance of COVID-19 vaccination was written by a lay person (i.e., ordinary people recounting their experience with the virus) or a medical expert did not affect intention [18].

#### 3.4.2. Chatbot

##### Vaccination Intention

A cross-sectional study (pre-print) found that a chatbot implemented via the most popular messenger app in Japan increased vaccination intentions. However, this was a low quality study with no control group and pre- and post-vaccine intentions were measured after the intervention [41].

#### 3.4.3. Video

##### Vaccination Behaviour

A field experiment (pre-print) found that adding educational videos to reminders to schedule COVID-19 vaccines did not increase the effectiveness of the reminders [16].

##### Vaccination Intention

There is mixed evidence regarding the effectiveness of videos in increasing vaccination intentions. An online experiment (pre-print) found that adding educational videos to reminders to schedule COVID-19 vaccines increased the effectiveness of a reminder and led to an increase in participants reported likelihood of scheduling an appointment [16]. An online RCT (pre-print) found that 22% of unvaccinated respondents expressed wanting further information on the vaccine after watching a video containing information on the health benefits of the vaccine and a cash voucher incentive for vaccination, compared to 14–16% for a video with lottery incentives or a standard information video without an incentive, respectively [19]. An online RCT (pre-print) found that only a male-narrated video providing details about the COVID-19 vaccine and emphasising the altruistic motivations associated with vaccination increased intention [42]. According to the authors, this could be due to politically conservative individuals within the sample who had lower vaccination intentions after a female-narrated video. However, the authors did not provide further information about controlling for extraneous differences between the narrator conditions, such as speaking style, which may have also accounted for the difference in effect. Finally, a study found that patients who watched a video discussing the COVID-19 vaccine were more likely to receive the COVID-19 vaccine than before watching the video, although intentions were only assessed after the intervention with no direct measure of pre-intervention intentions [43].

#### 3.4.4. Reminders

##### Vaccination Behaviour

Reminders increase vaccine uptake. A field RCT (pre-print) found that sending a second reminder to individuals eligible for vaccination who had not yet scheduled their first dose increased uptake [16]. Reminders with an emphasis on personalisation increased uptake, whereas adding an educational video did not increase uptake. Participants were then sent another reminder which increased uptake further, with all message types increasing uptake to a similar extent [16]. A field experiment (pre-print) found that sending a second reminder increased uptake, with the reminder emphasising personal benefits being more effective than the one emphasising social benefit [17].

##### Vaccination Intention

In an online experiment (pre-print), a text message booking reminder emphasising personalisation did not increase intention, unlike in the field RCT, of which it was a replication, where it increased uptake [16].

### 3.5. Policy

#### 3.5.1. Mandatory Vaccination

##### Vaccination Intention

The impact of mandatory vaccination depends on the initial vaccination intention. Two similar online RCTs found that there is high psychological reactance (i.e., negative emotions) to mandatory vaccination when individuals have a low intention to receive the vaccine [44]. One RCT also found that increased reactance led to higher intentions to avoid the COVID-19 vaccine, particularly for the mandatory condition. Participants were also more likely to take action (e.g., through activism) against the policy when the vaccine was mandatory, compared to unrestricted [44]. This supports research beyond the scope of this review, including a modelling study of COVID-19 vaccine uptake [45] and interviews with care home staff (pre-print) which suggests mandatory vaccination could lead to staff resignations [46].

#### 3.5.2. Vaccination Proof

##### Vaccination Intention

A DCE found that offering proof of vaccination increased vaccine uptake amongst individuals who want a vaccine card, compared to no proof, but it did not impact uptake amongst those who do not want proof [29].

#### 3.5.3. ‘Opt-Out’ Vaccination

##### Vaccination Intention

An online experiment found that pre-scheduling a vaccination appointment for an individual increased vaccine intention compared to asking participants if they wanted to receive the vaccine [47]. Another online study (pre-print) found that pre-selecting ‘yes’ or ‘no’ options as responses for wanting to receive the vaccine did not have an impact on vaccine acceptance [32].

#### 3.5.4. Prioritising Vaccination

##### Vaccination Intention

Two online RCTs reported that individuals with a high intention to receive the vaccine had high psychological reactance (i.e., negative emotions) when the vaccine is scarce and not widely available to them until 2022 [44].

#### 3.5.5. Legal Incentives

##### Vaccination Intention

An online RCT (pre-print) found that legal incentives, such as no longer being required to wear a face covering or to provide a negative test to access events, did not impact vaccination intention, compared to no legal incentive [48].

#### 3.5.6. Monetary Incentives

##### Vaccination Behaviour

Three quasi-experimental studies have found mixed results on the impact of monetary incentives on vaccination rates. When comparing the number of vaccine doses administered daily per 100,000 individuals between States with and without monetary incentives, one study (pre-print) found a decline in vaccinations between April and July 2021 in all States, irrespective of monetary incentive [49]. The authors suggest that as 40% of individuals were already fully vaccinated when incentives were introduced, small rewards or low-probability lotteries were perhaps insufficiently persuasive to the unvaccinated individuals.

Two studies have looked more specifically at the effectiveness of Ohio’s ‘Vax a Million’ programme, which awarded five people $1 million. One study found that vaccination rates, per 100,000 individuals, did not increase after the lottery was introduced [50]. They also found a decline in uptake in the rest of the US, but Ohio declined to a greater extent [50]. Another study (pre-print) found that Ohio did not have greater vaccination after the introduction of the incentive when comparing to an average of the US; but when using a synthetic control, they found that Ohio’s initiative did increase vaccinations [51]. They report that the incentive initiative encouraged 1.5% of Ohioans (i.e., 82,000 individuals) to receive the vaccine who would otherwise not be vaccinated [51].

##### Vaccination Intention

An online experiment found no effect of financial reward on willingness to be vaccinated [21], whilst another study (pre-print) found that a free of charge vaccine with a government subsidy reduced the likelihood of choosing to be vaccinated [30]. However, other evidence suggests that monetary incentives could potentially increase vaccine uptake, although the evidence is not clear on the optimum price. An online experiment (pre-print) found that monetary incentives above 3250 euros increased willingness to get vaccinated [48]. Incentives worked best amongst younger people and those who had less confidence in the vaccine and were more complacent about COVID-19. Similarly, another online experiment in the US found that vaccination intention increased by 4.5 percentage points from 70% with a $100 compensation and by 13.6 percentage points with $500 compared to $0 [47]. Importantly, small compensations backfired; the $20 compensation reduced intentions by 5 percentage points compared to no compensation. Finally, another German online experiment in November 2020 found no effect of financial reward on willingness to be vaccinated, after asking participants to imagine they had received a financial reward for getting vaccinated ranging between 25 and 200 euros, even after controlling for their financial situation [21].

While cash amounts could increase vaccination intentions, the evidence suggests that lotteries are less effective. An online RCT (pre-print) found no difference in expression of further interest in vaccination information between control and lottery conditions, but found that cash vouchers did increase the number of interested individuals [19]. Another online RCT (pre-print) compared 12 conditions varying the distribution of $5million and found that vaccination intention did not differ across conditions; indeed intentions post-incentive were strongly associated with baseline willingness [36]. In a second online RCT, this study altered the message framing of the lottery (e.g., gain vs. loss) and the numeric framing (e.g., 5 total winners vs. 1 winner for each of the 5 weeks) and again found no difference in vaccination intention [36].

#### 3.5.7. Cost

##### Vaccination Intention

Requiring people to pay for vaccines is likely to reduce vaccine uptake. One study (pre-print) found that charging HKD700 (£65) and HKD400 (£37) made the vaccines less likely to be chosen than a free vaccine [30].

### 3.6. Vaccination Delivery

#### 3.6.1. Setting

##### Vaccination Intention

A DCE found that nearby GP surgeries were the most preferred vaccination location, whilst a nearby pharmacy and drive-thru were the least preferred [39]. Additionally, an online experiment found that more participants chose to receive their vaccine in 3 weeks at their local GP compared to in 5 days in a mass vaccination centre [52]. However, more participants chose an earlier appointment at either a local GP or a mass vaccination centre rather than having a health care professional come to their home at a later date. In addition, a US discrete choice experiment suggests that preferred locations vary between individuals, which can impact uptake. Those who prefer a medical setting were less likely to choose a vaccine at a community-based vaccination centre, whereas those who prefer a community setting were less likely to choose a vaccine at a medical vaccination setting [29].

#### 3.6.2. Proximity

##### Vaccination Intention

A DCE found that a vaccination centre that was 15–30 min away was preferable to a centre less than 15 min away and 30–45 min away [39], which the authors posit could be because individuals had a specific vaccination centre in mind when considering the choices which were more likely to be 15–30 min away. Additionally, more respondents preferred to receive the vaccine within 3 weeks at a centre 10 min away compared to a centre 1 h away within 5 days [52]. This is supported by research (pre-print) published after this review was conducted which found an association between proximity of vaccine centre and vaccine uptake [8].

#### 3.6.3. Appointments

##### Vaccination Intention

A DCE found that weekday appointments after-hours were least preferred compared to a weekday 9 a.m.–5 p.m. or a weekend, although this had less of an impact on decision making than factors such as setting and proximity (as detailed above) [39]. An online experiment found that people preferred to wait 3 weeks for an appointment if they can choose their appointment time compared to receiving the vaccine within 5 days but not finding out their appointment time until the day [52]. This study also found that individuals preferred to book a specific timeslot, as opposed to attending a vaccination centre without pre-booking a specific time. However, as the walk-in centre was coupled with an appointment in 3 weeks, it is difficult to identify which factor was less desirable.

#### 3.6.4. Waiting Time

##### Vaccination Intention

A forced-choice study (pre-print) found that a wait of 7 and 14 days after registering to be vaccinated did not affect the likelihood of a vaccine being chosen, compared to no waiting time, but a delay of 30 days reduced the likelihood of a vaccine being chosen [30]. An online experiment identified that optimum waiting time is dependent on other components of the vaccine delivery process, including setting, proximity and control over appointment [52]. In particular, they found that two-thirds of people preferred to wait 3 weeks for an appointment, compared to 5 days, when vaccination sites are nearby, when one can choose their appointment time and when they can go to the local GP.

## 4. Recommendations

Recommendations for increasing COVID-19 vaccine uptake based on the findings of this review can be found in Table 2. The table specifies whether interventions have been tested on real behaviour and/or intentions, given that intentions do not necessarily translate into behaviour [53]. The average risk of bias assessment for each type of intervention is also provided (good, fair, and poor).

## 5. Future Implications

Vaccination is vital to protect the public against COVID-19. As vaccine roll outs continue across the world, both for primary and booster doses, it is important to understand the effectiveness of existing interventions aiming to increase COVID-19 vaccine uptake. This review shows that a broad range of interventions have been tested to increase the uptake of COVID-19 vaccination, with reasonable evidence to suggest that personalising communications and sending text message booking reminders are effective interventions. This has implications for local and national health authorities with regards to the way they communicate with the public regarding their eligibility for vaccination. The heterogeneity of results, particularly amongst vaccine hesitant compared to vaccine accepters, also highlights the importance of understanding an intervention’s intended audience, and their existing barriers. Although this has been explored in an intervention addressing safety and effectiveness concerns [25], and one addressing access barriers [52], more research is needed to identify effective and acceptable interventions for encouraging vaccine uptake amongst these groups in particular.

Finally, there were mixed findings for many of the interventions, particularly for studies measuring vaccination intention. Additionally, although much of the research used experimental designs, very few measured real behavioural outcomes, and therefore it is important that future research builds upon the current evidence to identify under which circumstances interventions are effective at increasing vaccine uptake. Understanding which interventions are most effective amongst vaccine-hesitant populations and in the context of booster vaccinations will be important as vaccine roll outs continue across the world.

Future research should also take into consideration the barriers which are unique to booster doses of the COVID-19 vaccine, such as previous experience with side-effects, lack of awareness of the necessity of booster doses and feeling misled regarding the benefits of the primary vaccine doses [70].

## 6. Limitations

This review examines the cutting-edge and evolving literature concerning COVID-19 vaccination to highlight possible interventions that could be used to increase uptake of the COVID-19 vaccine. Furthermore, the potential utility for this review to also influence the uptake of non-COVID-19 vaccinations moving forwards is undeniable. However, as with all research, there are limitations and caveats that must be borne in mind. First, although much of the research used experimental designs where the intervention was compared to either another intervention or a control group, very few measured scheduled appointments or actual vaccine uptake. Most focused on measures of intention or vaccine hesitancy rather than uptake data, and often involved online experiments presenting participants with hypothetical scenarios. Although this provides preliminary insight into the effectiveness of interventions, it does not always reflect real-life behaviours [16]. In addition, a minority of studies did not include a control group [32,43], so it is not possible to identify the extent of the intervention’s effectiveness.

It is also important to acknowledge that many studies sampled the general population, and therefore are likely to include individuals who already intend to receive the COVID-19 vaccine. Interventions need to tackle the barriers of groups who are more likely to be vaccine hesitant based on previous research, such as those living in a high deprivation area, some ethnic minority groups and those with lower reading ages [9]. Moreover, the studies included here took place at various points throughout the pandemic, which means that participants across studies could have had varying levels of pre-intervention knowledge and beliefs about COVID-19 and the vaccine, which could influence the effectiveness of an intervention [31]. This review also included studies from a range of countries, meaning caution should be used when applying findings from one country to another. Replicating possible interventions in the target country would be beneficial, preferably with well-designed randomised controlled field trials.

This review included studies measuring either general COVID-19 vaccination intention or behaviour or behaviour relating to receiving the first dose but was conducted before the subsequent booster vaccination roll out. While there is no a priori reason to expect that interventions reported herein would be widely ineffective if applied to the context of booster vaccination (indeed, some of the interventions identified above echo barriers and facilitators identified in the pre-COVID-19 vaccination literature [71,72] and the preliminary global insights on booster vaccinations [73,74]), further review work is recommended to bolster the evidence base for effective vaccination interventions. This is particularly pertinent given the novelty of COVID-19 booster vaccinations. Indeed, given the rapidly developing nature of the COVID-19 vaccine rollout and accompanying intervention design and evaluation, it will be important to continue to iterate this review beyond the end of the COVID-19 pandemic in order to capture best evidence for informing future vaccination programmes.

Finally, there are limitations relating to the conduct of this review. Given the range of interventions and outcomes included, it was not possible to provide effect measures for each study and compare these numerically or visually across studies. The two researchers assessing studies for inclusion discussed the set of studies initially identified as eligible after they each independently screened half of the studies. They did not double code or discuss the studies that were not deemed eligible. Formal methods of assessing heterogeneity between studies and confidence in the body of evidence were not used. This was to ensure this review was carried out in a timely manner and could adequately feed into COVID-19 vaccination programmes. Instead, the authors make clear in the table of recommendations (Table 2) which interventions lack clear evidence.

## 7. Conclusions

Across 39 studies, this systematic review shows that a broad range of interventions have been tested to increase the uptake of COVID-19 vaccination, tackling various aspects of communications, policy and delivery. We find reasonable evidence investigating real behaviour suggesting that personalising communications and sending text message booking reminders are effective. Findings on vaccination intention are mixed but suggest that communicating uncertainty about the vaccine does not decrease intention, whereas making vaccination mandatory could have a negative impact. However, the lack of evidence and the mixed findings in other areas warrant further research, ideally based on observed vaccination behaviour.

## Figures and Tables

**Table 1:** Characteristics of included studies.

Author	Design	Participants	Country	Intervention Type	Outcome	Comparison	Effectiveness	Risk of Bias
Barber and West	Quasi-experimental	Vaccination rates	US	Policy: monetary incentive	Behaviour	Synthetic control	Positive effect: lottery incentive increased vaccination uptake	Good
Bateman et al.	Online cross-sectional	*n* = 661 (patients)	UK	Communications—delivery: video	Intention	None	Positive effect: participants reported they were more likely to receive the vaccine after watching the video	Poor
Batteux et al.	Online RCT	*n* = 328 (general population)	UK	Communications—presentation: uncertainty	Intention	No additional comparison	Neutral effect: after the first announcement, there was no difference in vaccination intention between people who received the certain and uncertain announcement Positive effect: after the second announcement, participants who received the initial uncertain announcement had stronger vaccination intention	Fair
Berliner-Senderey et al.	Field RCT	*n* = 768,404 (unvaccinated over 16 s)	Israel	Communications—delivery: reminders Communications—content: benefits of vaccination, effectiveness and safety; social norms	Behaviour	No additional comparison	Positive effect: sending a text message reminder increased vaccination uptake Positive effect: messages focusing on personal benefit were more effect than messages focusing on social benefit	Fair
Behavioural Insights Team	Online experiment	*n* = 4085 (general population adults)	UK	Delivery: setting, proximity, appointments and waiting time	Intention	No additional comparison	Negative effect: more people chose the option to be vaccinated later, rather than sooner, when: 1. Travel 1 h vs. 10 min 2. Were told when to go vs. going at a time of choice 3. Going to a vaccination site vs. going to the GP	Fair
Chen et al.	Online RCT	*n* = 413 (general population)	China	Communications—presentation: framing, numerical format Communications—content: effectiveness and safety	Intention	No additional comparison	No effect of any news article on vaccination intention	Good
Craig	DCE	*n* = 1153 (general population)	US	Communications—content: effectiveness and safety Delivery: immunity Policy: proof of vaccination Vaccination delivery: Setting	Intention	No additional comparison	Negative effect: preference of vaccination was reduced when: 1. Duration of immunity was decreased 2. Vaccine effectiveness was reduced 3. Increasing risk of serious and adverse side-effects Mixed effects: negative effect of no proof of vaccination among those who want a vaccine, no effect for those who do not. There was also a negative effect of community setting among those who prefer a medical setting, and vice versa	Good
Dai et al.	Field RCT	*n* = 113,229 (patients registered at UCLA health)	US	Communications—delivery: reminders Communications—presentation: personalisation and video	Behaviour	No text message	Positive effects: sending a text message increased vaccine uptake and adding the ownership language increase uptake compared to the simple text No effect: no effect of adding a video compared to messages without a video	Good
	Field RCT	*n* = 90,662 (patients registered at UCLA health)	US	Communications—delivery: reminders Communications—content: benefits of vaccination Communications—presentation: personalisation	Behaviour	No text message	Positive effect: sending a second reminder increased vaccine uptake No effect: all message types increased vaccine uptake	Good
	Online RCT	*n* = 2003	US	Communications—content: personalisation Communications—delivery: Reminders communications—presentation: video	Intention	No text message	Positive effect: adding a video increased participants’ reported likelihood of scheduling a video No effect: adding ownership language did not increase participants’ reported likelihood of scheduling a video	Good
Davis et al.	Online RCT	*n* = 481 (vaccine-hesitant individuals)	UK	Communications—content: effectiveness and safety	Intention	No information	Positive effect: participants reported stronger COVID-19 vaccination intention when receiving information about COVID-19 vaccines compared to no information Positive effect: participants reported stronger COVID-19 vaccination information when receiving information about COVID-19 vaccines plus information describing 40% flu vaccine efficacy than participants who received only COVID-19 information	Good
Duch et al.	Online RCT	*n* = 1628 (unvaccinated US adults)	US	Communications—delivery: video Policy: monetary incentives	Intention	Health benefits of COVID-19 vaccine	No effect—lottery: no difference in percentage of people seeking more information when presented with a standard COVID-19 health information video compared to an information video plus information on a lottery Positive effective—cash voucher: more people sought more information after watching a video containing COVID-19 health information plus information on a cash-equivalent voucher	Good
Freeman et al.	Online RCT	*n* = 16,455 (general population)	UK	Communications—content: benefits of vaccination and vaccine development	Intention	NHS website information	No effect: no effects overall of any message type Positive effect: amongst strongly hesitant participants, messages describing personal benefits or safety concerns or combining the conditions reduced vaccine hesitancy compared to the control	Good
Han et al.	Online RCT	*n* = 1497 (general population)	US	Communications—presentation: uncertainty	Intention	Basic information about COVID-19	No effect: overall, no difference in intention between conditions Positive effective: higher vaccination intention when messages used uncertainty and uncertainty+normalising conditions	Good
Kerr et al.	Online RCT	*n* = 2488 (general population)	UK	Communications—content: effectiveness and safety	Intention	No information	No effect: no effect of messages conditions on vaccine hesitancy or vaccine intention	Good
	Online RCT	*n* = 2217 (general population)	UK	Communications—presentation: uncertainty	Intention	No additional comparison	No effect: no effect of message conditions on vaccine hesitancy or vaccine intention	Good
Kobayashi et al.	Cross-sectional	*n* = 10,192 (general population)	Japan	Communications—delivery: chatbot	Intention	None	Positive effect: vaccination intention increased after using the chatbot	Poor
McPhedran et al.	DCE	*n* = 2012 (18–29 unvaccinated adults)	UK	Delivery: setting, proximity, appointments Communications—presentation: messenger	Intention	No additional comparison	Positive effect: vaccinations were most preferred when: 1. Vaccinations were in a nearby GP surgery 2. Location proximity was 15–30 min away Negative effect: vaccinations were least preferred when: 1. Vaccinations were at a nearby pharmacy or drive-thru 2. Appointments were after hours in the week 3. Invitations were forwarded from one’s best friend 4. Location proximity was 30–45 min away	Good
Moehring et al.	Online RCT	*n* = 437,236 (general population)	23 countries	Communications—content: social norms	Intention	Delayed control	Positive effect: social norm framing increased vaccination acceptance	Good
Motta et al.	Online RCT	*n* = 7064 (general population)	US	Communications—presentation: messenger Communications—content: benefits of vaccination and vaccine development	Intention	Unrelated news story	Positive effects: vaccination intention increased for messages with a personal or collective frame, compared to the control and when no pre-bunking of clinical trials is included No effects: compared to the control, there was no effect of on vaccination intention when messages used an economic frame, lay person source, expert source or included pre-bunking of clinical trial information	Fair
Palm et al.	Online RCT	*n* = 1123 (general population)	US	Communications—content: effectiveness and safety, vaccine development and social norms Communications—presentation: messenger	Intention	No information	Positive effects: a news story describing that the vaccine is safe and effective or others being willing to get vaccinated increased vaccination intention compared to the control Negative effects: a news story describing that others are unwilling to get vaccinated or that Trump pushed approval decreased vaccination intention compared to the control No effects: a new story describing that the vaccine is unsafe and ineffective or a liberal mandatory agenda had no effect compared to the control	Good
Pink et al.	Online RCT	*n* = 1480 (Republicans)	US	Communications—presentation: messenger	Intention	A video and short essay on an unrelated topic	Positive effect: Republican endorsement was more effective than Democrat endorsement and the control message for unvaccinated participants	Good
Santos et al.	Field RCT	*n* = 9723 (health care workers)	US	Communications—content: social norms and effectiveness and safety	Behaviour	Delayed control	Positive effects: messages describing social norms and re-framing the vaccine risk both led to more vaccination registrations than the delayed control No effect: there was no difference in vaccination registrations between the two message types	Fair
Serra-Garcia and Szech	Online experiment	*n* = 1040 (targeting Black participants)	US	Policy: ‘opt-out’ vaccination and monetary incentives	Intention	Defaults: No additional comparison No compensation	Positive effects: stronger vaccination intentions for higher compensation and in the opt-out condition Negative effects: a smaller compensation decreased vaccination intend compared to no compensation	Good
Sinclair and Agerström	Online RCT	*n* = 654 (18–30 adults)	UK	Communications—content: social norms and effectiveness and safety	Intention	No information	Positive effect: participants reported stronger vaccination intentions for higher social norms than weaker social norms (85% vs. 45%) No effects: there was no difference in vaccination intention between both social norm messages and the NHS message. There was also no effect of norm group (young vs. general) on vaccination intention.	Good
Sprengholz et al.	Online RCT	*n* = 1349 (general population)	Germany	Policy: monetary incentive Communications—content: benefits of vaccination	Intention	A vaccine would be approved shortly No payment	No effects: there was no effect of communication or payment on participants’ reported likelihood of receiving the vaccine	Fair
Sprengholz et al.	Online RCT	*n* = 973 (general population)	Germany	Policy: Mandatory vaccination and prioritising vaccination	Intention	No additional comparison	Negative effects: high reactance to mandatory vaccination for those with lower a priori vaccination intention and a high reactance to scarcity of vaccination for those with higher a priori vaccination intention	Fair
	Online RCT	*n* = 1701 (groups not yet offered the vaccine)	US	Policy: mandatory vaccination and prioritising vaccination	Intention	No additional comparison	Negative effects: high reactance to mandatory vaccination for those with lower a priori vaccination intention and a high reactance to scarcity of vaccination for those with higher a priori vaccination intention	Good
Sprengholz et al.	Online experiment	*n* = 997 (general population)	Germany	Policy: legal and monetary incentives	Intention	No additional comparison	Positive effect: monetary incentives increased willingness to get vaccinated from 3250 euros onwards No effect: no impact of legal incentives (e.g., increased freedoms) on vaccination intention	Good
Strickland et al.	Online RCT	*n* = 497 (general population)	US	Policy: ‘opt-out’ vaccination	Intention	No pre-selection	No effects: no effect of pre-selecting ‘yes’ or ‘no’	Good
	Online experiment	*n* = 485 (general population)	US	Communications—content: vaccine development Communications—presentation: framing	Intention	No additional comparison	Positive effects: greater acceptance of less effective vaccines under a positive framing condition and when the vaccine was developed for 12 months	Good
Taber et al.	Online RCT	*n* = 589 (unvaccinated US adults)	US	Policy: monetary incentives	Intention	No additional comparison	No effects: vaccination intention did not differ across conditions	Good
	Online RCT	*n* = 274 (unvaccinated adults)	US	Communications—presentation: framing and numerical format	Intention	No additional comparison	No effects: vaccination intention did not differ across conditions	Good
Thirumurthy et al.	Quasi-experimental	Vaccination rates	US	Policy: monetary incentives	Behaviour	States with no incentive	No effect: no difference in vaccination trends between states and without incentives	Fair
Thorpe et al.	Online RCT	*n* = 1075 (general population)	US	Communications—content: effectiveness and safety, benefits of vaccination and vaccine development	Intention	No message	No effect: vaccination intention did not differ across conditions	Good
Trueblood et al.	Online RCT (Study 2)	*n* = 1003 (general population)	US	Communications—content: effectiveness and safety and herd immunity	Intention	Information about the vaccine approval process, side-effects and efficacy	Positive effect: when describing the necessary coverage to achieve herd immunity people were willing to receive the vaccine sooner than the control No effects: no differences in intention when comparing the COVID-19 vaccine efficacy to the flu relative to the control and when combining information on flu comparison and describing necessary coverage compared to the control	Good
Walkley et al.	Quasi-experimental	Vaccination rates	US	Policy: monetary incentives	Behaviour	Prior to incentive and states with no incentive	No effect: no effect of lottery-based incentive in Ohio on vaccination uptake	Good
Witus and Larson	Online RCT	*n* = 1632 (general population)	US	Communications—delivery: Video	Intention	No information	Positive effect: higher vaccination intention amongst those watching the male-narrated video compared to the control group No effects: no difference in vaccination intention between the female-narrated video and the control and the blog post and the control	Fair
Yuen et al.	Online cross-sectional	*n* = 2733 (adults above 18)	Hong Kong	Policy: monetary incentives and cost Communications—content: effectiveness and safety Delivery: waiting time	Intention	No additional comparison	Positive effects: vaccines were more likely to be chosen when: 1. They had 95% efficacy compared to 50% or 70% efficacy. 2. Medical insurance is provided against severe side-effects compared to not provided Negative effects: vaccinations were less likely to be chosen when: 1. Subsidies were provided (compared to a free vaccines) 2. There is a wait of 30 days (compared to no waiting time) No effects: vaccinations were less likely to be chosen when: 1. Increased likelihood of mild side-effects 2. Increased likelihood of severe side-effects 3. Subsidies were provided (compared to a medical insurance) 4. There is a wait of 7 or 14 days (compared to no waiting time)	Good

**Table 2:** Recommendations for increasing vaccination.

Intervention	Recommendation
*Communications content* Benefits of vaccination	**Recommendation:** Emphasising the benefits of vaccination to the self and others can be effective, but there are mixed findings. Studies on behaviour find that it is not more effective than informing individuals of where they can receive the vaccine [16]. There is evidence from studies on intention that it could be more effective with strongly hesitant groups, although it might not affect all demographic groups in the same way [20]. One possible explanation is that people already know the benefits and so there are other barriers to vaccination that are reducing uptake/ intentions. Indeed, more co-production work is likely to be beneficial in helping to identify the benefits relevant to specific groups. This use of co-production can also help identify how the benefits should be communicated with the target population (e.g., using appropriate content, communication sources or messengers). **Outcome:** Behaviour and intention **Overall quality:** Good/Fair (7 studies)
*Communications content* Effectiveness and safety	**Recommendation:** Reminders that emphasise vaccine effectiveness can increase vaccination uptake [17]. Evidence on intention suggests that communicating the effectiveness and safety of vaccines can have a positive effect, and could in fact be stronger for vaccine-hesitant individuals [25]. However, the evidence is mixed, with some research suggesting that it is no more effective than a control. The evidence also suggests that describing effectiveness and safety is more effective when there is high vaccine effectiveness and low risk of side-effects [29,30]. **Outcome:** Behaviour and intention **Overall quality:** Good (11 studies)
*Communications content* Vaccine development	**Recommendation:** It is unclear whether addressing the speed of development of vaccines and robustness of trials affects uptake since evidence is mixed [18]. However, one trial suggests that it could decrease hesitancy amongst vaccine-hesitant individuals [20]. **Outcome:** Intention **Overall quality:** Good (5 studies)
*Communications content* Social norms	**Recommendation:** Booking reminders telling others to ‘join the millions’ can be effective at increasing vaccine uptake but likely to be less effective than messages emphasising other aspects such as personal benefits [17] and safety [23]. Communicating that others intend to get vaccinated elicits mixed findings on intention, although could be effective in contexts where vaccination intentions are not clear [33], such as booster vaccinations. Social norms are a key facilitator for engaging in a range of behaviours and social norm interventions can be successful [54]. To be effective, social norms interventions need to be tailored to the target group, delivered by a member of the target group or a trusted individual and relevant to existing group norms [55]. **Outcome:** Behaviour and intention **Overall quality:** Good/Fair (5 studies)
*Communications content* Herd immunity	**Recommendation:** Highlighting how many need to be vaccinated to reach herd immunity could be effective, although this evidence is from one study [18]. Previous experimental evidence also suggests that communicating about herd immunity can increase vaccine uptake [34,35]. **Outcome:** Intention **Overall quality:** Good (1 study)
*Communications presentation* Personalisation	**Recommendation:** Vaccination invitations that mention that a vaccine has been made available to them can be effective in increasing uptake [16]. However, when sending a second reminder, personalisation is comparable to reminders [16]. Contrary to the field trial, an identical online experiment found that telling individuals a COVID-19 vaccine has been made available to them did not increase intention [16]. **Outcome:** Behaviour and intention **Overall quality:** Good (3 studies)
*Communications presentation* Framing	**Recommendation:** The effects of positive relative to negative framing are unclear from this limited evidence, suggesting that neither positive nor negative framing is more effective. A meta-analysis of framing effects in other vaccinations suggest that there is no effect of framing on vaccine intention [56], and therefore both positively and negatively framed information could be used. **Outcome:** Intention **Overall quality:** Good (3 studies)
*Communications presentation* Numerical format	**Recommendation:** The format in which effectiveness or lottery outcomes is communicated had no effect on vaccination intentions [31,36], although previous studies suggest frequencies are easier to understand than probabilities [57,58]. **Outcome:** Intention **Overall quality:** Good (2 studies)
*Communications presentation* Uncertainty	**Recommendation:** Communicating uncertainty about COVID-19 vaccines does not seem to decrease vaccination intention and may even be protective in terms of maintaining vaccination intention and trust in communicators if conflicting information arises over time [27,38]. This is consistent with guidance from the British Psychological Society, which recommends to communicate uncertainty and acknowledge change [59]. **Outcome:** Intention **Overall quality:** Good/Fair (3 studies)
*Communications delivery* Messenger	**Recommendation:** Match the messenger to the receiver in terms of characteristics, views, etc., as indicated by previous research [60,61]. Avoid controversial figures that might be divisive [26] and use trusted sources (e.g., NHS and GP). These trusted sources may vary between groups, meaning it is critically important to understand the views of target groups and their relationship to different sources. **Outcome:** Intention **Overall quality:** Good (4 studies)
*Communications delivery* Chatbot	**Recommendation:** Having an automated and instant chatbot providing vaccine information could increase vaccine uptake, although evidence is from one low-quality study with no control group and pre- and post-intervention vaccination intentions measured post-intervention [41]. **Outcome:** Intention **Overall quality:** Poor (1 study)
*Communications delivery* Video	**Recommendation:** Adding educational videos to reminders does not increase the effectiveness of the reminder in increasing vaccine uptake. There is mixed evidence regarding the use of videos in increasing vaccine intention. There are also difficulties with ensuring the videos reach a wide audience [43]. **Outcome:** Behaviour and intention **Overall quality:** Good/Fair (4 studies)
*Communications delivery* Reminders	**Recommendation:** Sending text message reminders increases vaccine uptake, as is the case in the interventions for the influenza vaccine [62]. **Outcome:** Behaviour and intention **Overall quality:** Good (4 studies)
*Policy* Mandatory vaccination	**Recommendation:** Mandating vaccination is unlikely to be an effective strategy to increase vaccination uptake, particularly amongst people who already have low intentions to receive the vaccine [44]. Mandating vaccinations could also lead to a reduction in uptake of future doses [63], resignation of health care staff [46] and exacerbating inequalities through increased risk of enforcement [64]. **Outcome:** Intention **Overall quality:** Fair/Good (2 studies)
*Policy* Vaccination proof	**Recommendation:** One study suggests that proof of vaccination (e.g., vaccination card) should be offered [29], although other literature suggests negative public attitudes towards requiring proof of vaccination for domestic activities and also a possible reduction in uptake, particularly amongst those who are vaccine hesitant [65,66]. **Outcome:** Intention **Overall quality:** Good (1 study)
*Policy* “Opt-out” vaccination	**Recommendation:** There is some evidence to suggest that automatically opting people into vaccine, such as pre-scheduling vaccine appointments, could be effective [47]. However, the manipulations within these experiments are not a true reflection of the design or impact of an opt-out vaccination system. **Outcome:** Intention **Overall quality:** Good (2 studies)
*Policy* Prioritising vaccination	**Recommendation:** Holding back or limiting vaccines could reduce uptake in individuals who are highly motivated to receive the vaccine [44]. **Outcome:** Intention **Overall quality:** Fair/Good (2 studies)
*Policy* Legal incentives	**Recommendation:** There is no evidence that offering easing of restrictions (i.e., face covering or testing) increases vaccine uptake, although this was only from one study in Germany [48]. **Outcome:** Intention **Overall quality:** Good (1 study)
*Policy* Monetary incentives	**Recommendation:** The limited evidence on monetary incentives for uptake in the US is mixed. There is some evidence to suggest offering monetary incentives is effective on intention, although small amounts could backfire [49]. Caution should therefore be applied when considering any monetary incentive. Offering a low incentive could reduce uptake amongst individuals with intrinsic, altruistic motivations to have the vaccine, whereas offering a large incentive could be deemed uneconomical and coercive [21,67]. Additionally, while a fixed sum of money or cash-equivalent vouchers could be effective, lotteries may not be effective [19]. **Outcome:** Behaviour and intention **Overall quality:** Good (10 studies)
*Policy* Cost	**Recommendation:** Requiring payment, including with a subsidy, for vaccination is likely to reduce uptake [30]. **Outcome:** Intention **Overall quality:** Good (1 study)
*Vaccination Delivery* Setting	**Recommendation:** Offer vaccinations in both community (e.g., pharmacy, local supermarket, or workplace) and medical settings (e.g., GP, hospital, and clinic) [29]. These settings should also be easily accessible by public transport [52,68]. **Outcome:** Intention **Overall quality:** Good/Fair (3 studies)
*Vaccination Delivery* Proximity	**Recommendation:** Limited evidence suggests that vaccine centres more than 30 min away could reduce uptake to vaccination [52]. **Outcome:** Intention **Overall quality:** Fair/Good (2 studies)
*Vaccination Delivery* Appointments	**Recommendation:** There is some evidence that appointments during the working day are preferred, although these factors are less influential than setting and proximity [39]. There is not enough evidence to determine the extent to which pre-booking appointments is preferable to walk-in centres, although it is possible that walk-in centres are more suitable for particular groups (e.g., traveller communities, homeless individuals) [69]. It is also important that booking systems work efficiently, as difficulties with the booking process, such as website crashes and telephone queues, are barriers to uptake [5]. **Outcome:** Intention **Overall quality:** Fair/Good (2 studies)
*Vaccination Delivery* Waiting time	**Recommendation:** Vaccine appointments not available within 30 days could discourage uptake, although this evidence is only from one study [28]. Individuals might be more willing to wait for an appointment which is nearer and which they can choose the time of [52]. **Outcome:** Intention **Overall quality:** Fair/Good (2 studies)

## Data Availability

Not applicable.

## Supplementary Materials

The following supporting information can be downloaded at: https://www.mdpi.com/article/10.3390/vaccines10030386/s1, Table S1: PRISMA 2020 checklist; Table S2: Search terms; Table S3: Table of included studies; Table S4: Risk of bias.
